# Acute Myringitis—Atropine in Its Treatment

**Published:** 1873-01

**Authors:** A. D. Williams

**Affiliations:** (Late of Cincinnati), St. Louis, Mo.


### ACUTE MYRINGITIS—THE USE OF ATROPINE IN ITS TREATMENT.

BY A. D. WILLIAMS, M.D., (LATE OF CINCINNATI), ST. LOUIS, MO.

Myringitis is an inflammation of the membrana tympani. It comes on, as a rule, suddenly, and mostly at night. The patient is suddenly aroused from his slumbers by the severe pain, and, for him, there is no more sleep till the acute myringitis is gone. If anybody wishes to know what pain, *pure* pain, is, let him have an attack of acute inflammation of the membrana tympani. I think I have seen as severe suffering from it as from any other known cause. Fortunately, it is not common; but anybody may have it. Persons whose health is below par are most likely to have it.

Exposure in cold weather, and in strong wind, is, perhaps, its usual exciting cause. Sometimes it comes on apparently without any immediate cause. Why it should make its first appearance at night, usually, is impossible to say. The severe pain in and around the ear, extending to the whole side of the head, and, possibly, to the entire head, is its chief subjective symptom. The *pain* is the great trouble. It is worse at night, but does not cease in daytime. There is always more or less *dullness* of hearing, if not *actual* deafness. The patient very often complains of the sound of his own voice, ringing through the affected ear, every time he speaks, in a very unpleasant way. It is very annoying, and is usually compared to the sound produced by speaking into the end of an empty barrel. In my experience, this ringing is *particularly* indicative of *simple* or uncomplicated myringitis. The membrana tympani, very soon after the attack, loses its natural pale red glistening surface, and becomes intensely red. In the very incipient stage, the individual blood vessels may be seen, but these are soon lost in the general redness. The handle of the malleus, and the cone of light, very soon disappear, in consequence of the general swelling of the inflamed membrane, the surface of which becomes even, and has a flesh-like appearance. On account of these changes, it is sometimes difficult to recognize the membrane. In simple or uncomplicated cases (not preceded by some other disease), the inflammation is confined to the membrana tympani. It does not extend to the external meatus, nor to the cavity of the tympanum. This is a singular fact, considering the very intimate anatomical relations of the membrane to the surrounding parts. My experience, in closely observing several cases, shows it to be a fact, however.

There are other symptoms, but I have given the most prominent ones.

*Treatment.*—I desire particularly to call attention to what I think is the best treatment of acute myringitis. I will suppose that I

have a well-marked case of the disease before me. There is no question as to the diagnosis. How shall I treat it? That is the most important matter in all diseases. To the exclusion of everything else, I will prescibe an atropine solution (four to six grains to the ounce of water), to be dropped into the ear every hour till the pain is relieved, and three or four times a day afterward. It is best to warm the solution, so it will not feel too cold in the ear. I will expect this treatment to give perfect relief from the intense pain in a very short time after its first application. And in the course of ten days to two weeks, I will expect the ear to be restored to its normal condition. From my observations, atropine may be considered as a *specific* for acute myringitis. Its good effect is immediate and effectual. Exactly how it acts I do not know; but I presume its virtue lies mainly in its powerful anodyne effect. This anodyne property gives relief to the suffering, and, at the same time, has a decidedly antiphlogistic action.

Anodynes, in general, are not known to be antiphlogistic remedies. By this I mean to say that they have a favorable action on inflammations.

Under the use of atropine, the membrane assumes, very rapidly, its normal appearance.

I do not mean to say that all painful affections of the ear will be instantly cured by the use of atropine. A *correct diagnosis* (not always easy) must be made, and every case of idiopathic, uncomplicated, acute myringitis will be almost instantly relieved by the use of atropine. That has been the result in all the cases I have treated with it. As I said before, such cases are not common, but persons who have a large ear practice will meet them occasionally.

I refer only to one case in detail here. Some years since, at Cincinnati, I treated an old lady, the particulars of whose case were as follows:

Mrs. ——, aged forty-seven, was not robust naturally, and much reduced by the long-continued, fearful suffering. Six weeks ago she was suddenly waked up at night with intense pain in one of her ears. She declared she had not slept a wink for the last six long weeks, and begged piteously for relief from the pain, if it was only for a short time, that she might get a little rest—a few hours of sleep. The membrane presented the characteristic appearances above described; was intensely red; outlines all obscured, and looked more like a smooth piece of flesh. The diagnosis was acute myringitis, without complication.

I prescribed atropine (four-grain solution) to be dropped into the ear every hour till the pain was relieved, and then about five times a day, and at night, if necessary.

The first application relieved the fearful pain she had suffered for six weeks, as she afterward stated, in the course of an hour. That night she slept soundly all night. The atropine was continued from three to five times a day till she was dismissed as well. The membrane rapidly regained its normal appearance, and in about ten days she was discharged from further treatment. The atropine must have done the work, as nothing else was used in her case.

I give this as a typical case of acute myringitis, and of the very favorable effects of atropine upon it. I could give other similar cases, but this one will suffice.

There are no unpleasant effects from the use of atropine in the ear of grown persons. I have used even the atropine in substance. In children caution should be exercised.

I *accidentally* prescribed the atropine in the first case of acute myringitis in which I used it. Since then, I don't think of using anything else. For me, at least, it has worked like a *charm.* It is a useful remedy in other painful affections of the ear.

723 *Chestnut street.*

[Since this communication was in the hands of the compositor, I have had a case of the affection described, occurring in a strong, robust young man, about twenty-two years of age, in which the solution of atropine afforded the most satisfactory results. The attack came on suddenly, at night, following exposure to a cold, damp wind through the day. He described the pain as *in the ear*, and most intense, with a sensation of fullness, and entire deafness, the pain also extending for some distance around the ear. The relief from the atropine solution, with a brisk cathartic—the latter given on general principles, the bowels being constipated—was so effectual that, on the afternoon of the second day, although the deafness still continued, he, contrary to my instructions, resumed his avocation as driver of a light wagon. During the following night the pain suddenly returned, and with increased intensity, involving not only the ear, *deep in*, as he described it, but also the entire side of the head and face and muscles of the neck, so that it pained him to speak. This pain was so intolerable that he was brought to my residence, a number of squares, by a companion, "for more of the drops." A reäpplication of the atropine solution again afforded prompt relief—"in less than ten minutes all the pain was gone"—and under its use he continues comfortable, but the fullness and deafness still continue. The relief afforded in this case has certainly been most satisfactory, both to the patient and myself.—Ed.]—*Medical Archives.*

